# Skeletal muscle metabolism in rats with low and high intrinsic aerobic capacity: Effect of aging and exercise training

**DOI:** 10.1371/journal.pone.0208703

**Published:** 2018-12-11

**Authors:** Mingshu Shi, Øyvind Ellingsen, Tone Frost Bathen, Morten A. Høydal, Lauren G. Koch, Steven L. Britton, Ulrik Wisløff, Tomas O. Stølen, Morteza Esmaeili

**Affiliations:** 1 Department of Circulation and Medical Imaging, Norwegian University of Science and Technology, Trondheim, Norway; 2 Clinic of Cardiology, St Olavs Hospital, Trondheim, Norway; 3 Clinic of Cardiothoracic Surgery, St Olavs Hospital, Trondheim, Norway; 4 Department of Physiology and Pharmacology, The University of Toledo, Toledo, Ohio, United States of America; 5 Department of Anesthesiology, University of Michigan, Ann Arbor, Michigan, United States of America; 6 Department of Molecular and Integrative Physiology, University of Michigan, Ann Arbor, Michigan, United States of America; 7 School of Human Movement & Nutrition Sciences, University of Queensland, St.Lucia, Queensland, Australia; University of Tennessee Health Science Center College of Graduate Health Sciences, UNITED STATES

## Abstract

**Purpose:**

Exercise training increases aerobic capacity and is beneficial for health, whereas low aerobic exercise capacity is a strong independent predictor of cardiovascular disease and premature death. The purpose of the present study was to determine the metabolic profiles in a rat model of inborn low versus high capacity runners (LCR/HCR) and to determine the effect of inborn aerobic capacity, aging, and exercise training on skeletal muscle metabolic profile.

**Methods:**

LCR/HCR rats were randomized to high intensity low volume interval treadmill training twice a week or sedentary control for 3 or 11 months before they were sacrificed, at 9 and 18 months of age, respectively. Magnetic resonance spectra were acquired from soleus muscle extracts, and partial least square discriminative analysis was used to determine the differences in metabolic profile.

**Results:**

Sedentary HCR rats had 54% and 30% higher *V*O_2max_ compared to sedentary LCR rats at 9 months and 18 months, respectively. In HCR, exercise increased running speed significantly, and *V*O_2max_ was higher at age of 9 months, compared to sedentary counterparts. In LCR, changes were small and did not reach the level of significance. The metabolic profile was significantly different in the LCR sedentary group compared to the HCR sedentary group at the age of 9 and 18 months, with higher glutamine and glutamate levels (9 months) and lower lactate level (18 months) in HCR. Irrespective of fitness level, aging was associated with increased soleus muscle concentrations of glycerophosphocholine and glucose. Interval training did not influence metabolic profiles in LCR or HCR rats at any age.

**Conclusion:**

Differences in inborn aerobic capacity gave the most marked contrasts in metabolic profile, there were also some changes with ageing. Low volume high intensity interval training twice a week had no detectable effect on metabolic profile.

## Introduction

Low exercise capacity measured by maximal oxygen consumption (*V*O_2max_) is an independent predictor of premature death, stronger than other established risk factor [1–4]. Hence it is often assumed that exercise training that increases aerobic capacity will improve health outcomes. Comprising the largest organ system in the human body, skeletal muscle metabolism profoundly impacts whole-body nutrient homeostasis [5]. Low exercise capacity is highly correlated with skeletal muscle dysfunction and metabolic disorders, such as obesity, diabetes, and cardiovascular disease [6–8]. However, aerobic capacity not only depends on physical activity, but on its interaction with aging and genotype. As discussed below, skeletal muscle function and metabolism are influenced by genotype, aging and exercise training in a complex way. However, the interplay between these factors is not well characterized.

Genotype determines as much as 50% of differences in *V*O_2max_ among individuals [9]. In a rat model bred for low versus high inborn running capacity (LCR/HCR) [10], LCR rats had fewer capillaries in the soleus muscle and lower levels of intramuscular glycogen and mitochondrial content compared to HCR. Genes associated with skeletal muscle, mitochondrial function, and oxidative energy metabolism were differentially expressed between LCR and HCR [10–12].

Aging leads to a loss of muscle mass and a decline in skeletal muscle function [13]. This is associated with an imbalance between protein synthesis and protein break down, and with impaired protein and amino acid metabolism [14]. Furthermore, the balance between glucose and lipid metabolism is changed [15]. Combined, these changes predispose for sarcopenia, insulin resistance, impaired glucose tolerance, and metabolic syndromes [14].

Exercise training is regarded as the most effective method to increase muscle performance and metabolism. Especially high intensity exercise training has proven to be effective [16, 17]. Key regulators such as AMPK and PGC-1α are activated, thus increasing mitochondrial oxidative respiration and biogenesis [18–20]. However, it is not known whether low volumes of exercise training at high intensity might influence metabolism in the context of ageing and different genetic backgrounds.

To get a better understanding of the interplay between aerobic capacity and skeletal muscle metabolic profile, the effect of aging and low volume exercise training were assessed in the experimental LCR/HCR rat model. [1, 21]. We hypothesized that age, intrinsic running capacity, and exercise training would affect skeletal muscle metabolic profile, and that the changes would be related to aerobic energy metabolism and share similarities with the pathogenesis of the metabolic syndrome [22–25]. A long-term goal was to find a novel method to identify impaired skeletal muscle metabolism by using metabolomics based on magnetic resonance spectroscopy (MRS) and *V*O_2max_.

## Materials and methods

### Ethical perspective

All animal studies were approved by the Norwegian Council for Animal Research, and conform to the *Guide for the Care and Use of Laboratory Animals* published by the US National Institutes of Health (NIH Publication No. 85–23, revised 1996). Each participant had protocols for animal research approved by their National Council for Animal Research. The experiments were designed according to the guidelines from the Federation of European Laboratory Animal Science Associations (FELASA), EU animal research directive (86/609/EEC) and Council of Europe (ETS 123), and the EU directive from 2013 (2010/63/EU). All researchers in this study have a FELASA C certificate. Animal caretakers at the animal facility have either a FELASA C or FELASA B certificate. In addition to daily supervision by the animal caretakers, the veterinarian examines all animals once a week and is available for consult every day. The 3 R’s (Replacement, Reduction and Refinement) have specifically been addressed when designing the study.

### Rat model of intrinsic running capacity

The LCR and HCR models were established by Koch and Britton in 2001 [21]. Briefly, the rat founder population originated from the genetically heterogeneous N:NIH outcrossed stock [26]. Running capacity was determined by a ramp treadmill running protocol until exhaustion. A rotational breeding scheme was performed to extend the possibility of a varied allelic combination. The rats used in the current study were from generation 29 and 30.

### Exercise training and *VO*_2max_ testing

Aerobic fitness was quantified as *V*O_2max_ normalized to scaled body mass. We used an individualized protocol as previously described [27]. Briefly, each rat had a 10-min warm-up at slow to medium pace based on previous experiments, before the *V*O_2max_ test. The test was performed on a treadmill in a closed chamber customized for rats (Columbus instruments, USA). Oxygen concentrations in and out of the chamber were measured and airflow through the chamber was controlled by an in-house build system. Band speed was increased by 1.8 m/min every 2 minute until the rat was unable to maintain the running speed, which was recorded as the max running speed for rats. After the *V*O_2max_ test, HCR and LCR rats were randomized to four subgroups (6 in each group): HCR rats with exercise training (HCR trained) or sedentary (HCR sed), LCR rats with exercise training (LCR trained) or sedentary (LCR sed). Training was conducted 60 min/day, 5 days/week, for 6 weeks. After the first 6 weeks, rats exercised two times per week throughout the study period. The high-intensity exercise training started with a 10 min warm-up at 50–60% of *V*O_2max_. Thereafter, rats ran 10 times 4 min at 80%-90% of *V*O_2max_, separated by 2 min active breaks at 50% intensity. *V*O_2max_ was measured at baseline, after 3 months (9 months of age) and 12 months (18 months of age) 5 days before sacrifice and tissue collection. The 9 months old rats then exercised the first 6 weeks with 5 sessions per week followed by 6 weeks with 2 sessions per week. The 18 month old rats exercised the first 6 weeks with 5 sessions per week and thereafter 46 weeks with 2 sessions per week.

### Tissue extraction

One day after the final exercise session (and at least 5 days since the latest *V*O_2max_ test), rats were anesthetized with 5% isoflurane, intubated and ventilated with 1.5% isoflurane in a 70% O_2_/30% N_2_O. The soleus muscles were quickly removed and placed in ice-cold saline for dissection. Performing an identical surgical procedure and by snap-freezing, the variation in surgical time for tissue resection was minimized among the subgroups. Frozen tissues were extracted using a modified dual phase extraction protocol [28]. Briefly, the muscle samples were powdered in a morter with liquid N_2_ and transferred to a 2 mL cryotube. Next, we added methanol (two times the tissue weight in mg), 150 μL purified water, and chloroform (1.5 times of the tissue weight) to the sample. After centrifuging, the upper layer water phase was transferred to a new tube, frozen at -80 °C, lyophilized, and stored at 4°C until MRS analysis.

### Proton MR spectroscopy

Before MRS analysis, samples were dissolved in deuterium oxide (D_2_O, Sigma-Aldrich Corporation, USA). The pH of all samples was adjusted to the same level (pH ~ 7) by perchloric acid and potassium hydroxide. MR spectroscopy was performed using a Bruker Avance III Ultrashielded Plus 600 MHz spectrometer (Bruker Biospin GmbH, Germany) equipped with a 5 mm QCI Cryoprobe with integrated, cooled preamplifiers for ^1^H, ^2^H, and ^13^C. This MR system provided a fully automated experiment in combination with Icon-NMR on TopSpin v3.1 software (Bruker Biospin). The MR spectra were obtained at 28.05 °C using a standard protocol [29], for proton one-dimensional nuclear Overhauser effect spectroscopy (1D-NOESY) (noesygppr1d; Bruker) with the following acquisition parameters: 128 scans, acquisition time of 2.73s, relaxation delay of 4s, free induction decay (FID) size of 65536, mixing time of 10ms, spectral width of 20.0243 ppm, and a total scan time of 349s.

### Data processing and multivariate analysis

MR spectra were automatically Fourier transformed with an exponential line broadening of 0.3 Hz, phased, and baseline corrected in Topspin. Pre-processed spectra were transferred into MATLAB R2013b (The Mathworks, Inc., USA) and referenced to the TSP peak at 0 ppm before peak alignment. Two low-quality spectra with poor water suppression and poor shim were removed from further analyses. Chemical shift differences were corrected by Icoshift algorithm [30]. Metabolites were assigned using NMR Suite 7.5 software (Chenomix Inc., AB, Edmonton, Canada). The area under the curve (AUC) of individual metabolite peaks were calculated using MATLAB. Prior to integration, the spectra were binned (bin size 0.01 ppm) and normalized by total area [31]. AUCs of individual metabolites were used as input variables for multivariate analysis. Multivariate analysis was performed in MATLAB with PLS Toolbox 8.0.2 (Eigenvector Research Inc., WA, USA). After auto-scaling the variables, a partial least square discriminant analysis (PLS-DA) models [32] were built to discriminate between age-groups (9 months and 18 months), intrinsic running capacities (LCR and HCR), and exercise training (sedentary and trained). The model was cross-validated by the Venetian blinds method and the leave-one-out method according to the sample size of the groups. Metabolites with variable importance to projection (VIP) scores of greater than 1 were determined as major contributors to the discrimination [33]. Orthogonal PLS (OPLS) was used to optimize the model if the number of latent variables (LV) was greater than 1. A permutation test was performed by using the self-predicted Wilcoxon signed rank test, and the difference was considered significant if the *P* value was <0.05.

### Univariate analysis

Univariate analysis was performed in GraphPad Prism 7.0 (GraphPad Software, San Diego, CA, USA) and R version 3.4.1. Three-way ANOVA was used to assess the effect of age, intrinsic running capacity, and exercise training on *V*O_2max_ and running speed. Multiple comparisons were performed by a Tukey pairwise multiple comparisons procedure. The comparisons of metabolite levels were performed by student’s t test. The difference was considered significant if the *P* value was <0.05.

## Results

### Cardiorespiratory fitness

**Fig 1:**
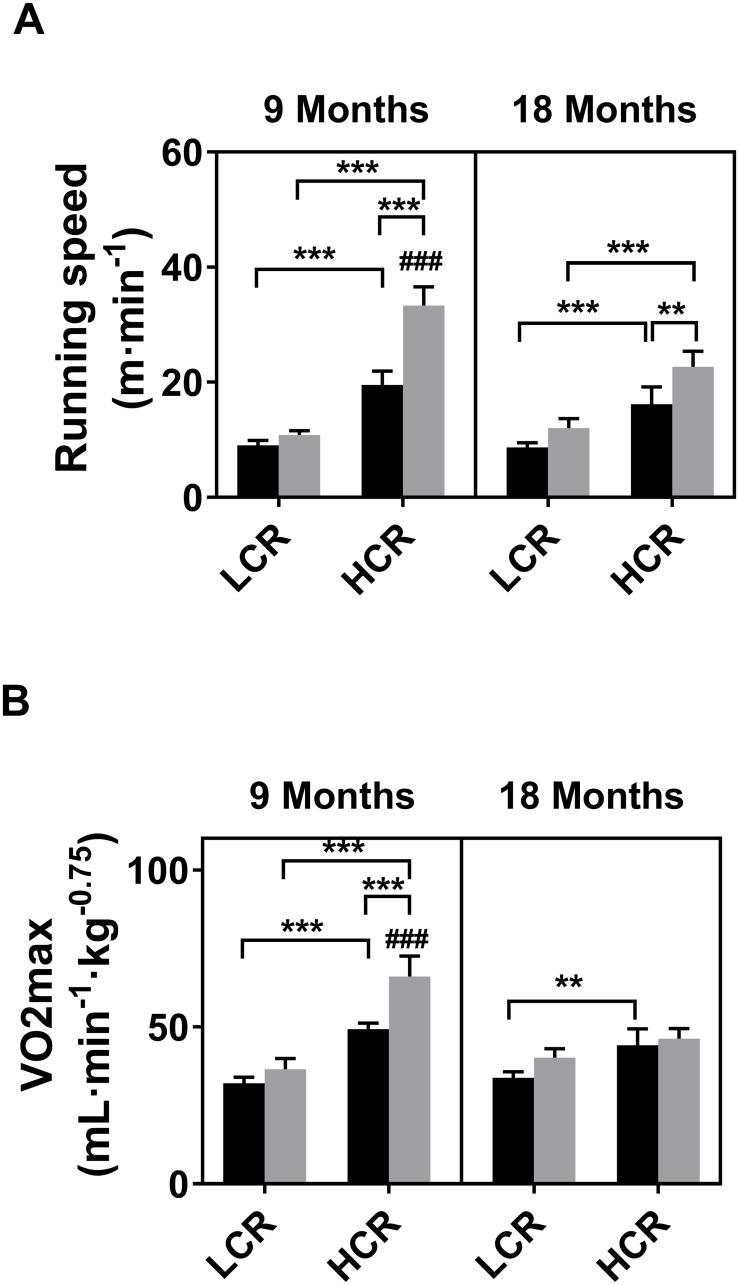
Effect of exercise training in aging LCR/HCR rats. Black and grey columns indicate sedentary and trained groups, respectively. **Panel A:** Running speed. **Panel B:** Oxygen consumption expressed in volume per minute, normalized to scaled body weight. *P* values: **, <0.01; ***, <0.001; ^###^, <0.001, different from respective HCR at 18 months.

Significant differences in running speed and aerobic capacity were observed in the LCR/HCR experimental model. As expected, running speed was lower in LCR than in HCR at 9 and 18 months of age (Fig 1, S1 Table). Similar effects were found with *V*O_2max_, except in the training group at 18 months. Pairwise comparisons revealed that exercising HCR rats had higher running speed compared to their sedentary counterparts at 9 and 18 months of age. Exercising HCR rats also had higher *V*O_2max_ than their sedentary counterparts at 9 months, but not at 18 months. In LCR, there were no significant changes with ageing or exercise.

### Metabolic profiles of skeletal muscle

**Fig 2:**
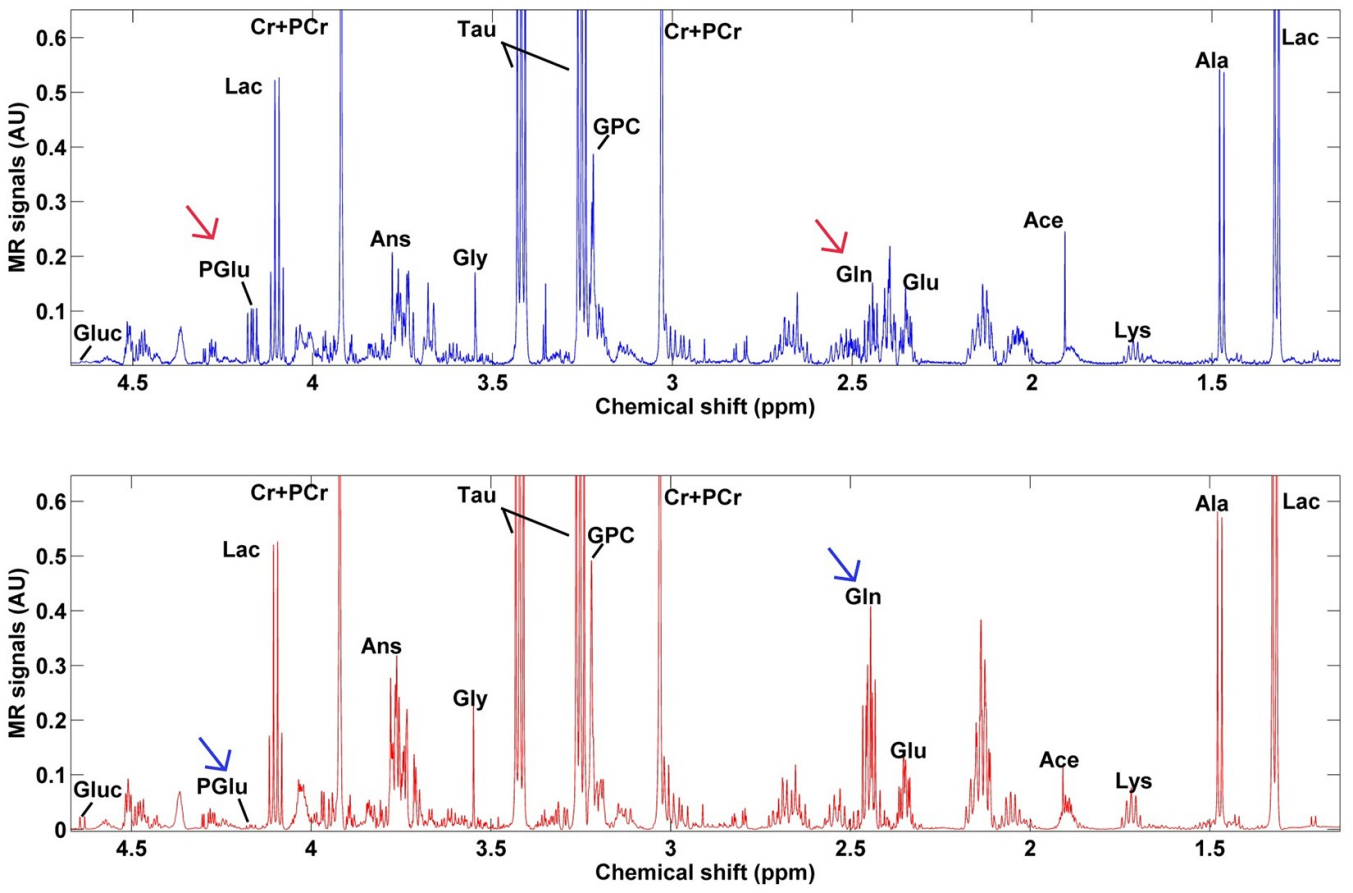
Proton MR spectra in soleus muscle from sedentary rats at 9 months of age. Spectra in figure show mean values of all measurements in either group. Upper panel: HCR; Lower panel LCR. Metabolite labeling: Lac, lactate; Ala, alanine; Lys, lysine; Ace, acetate; Glu, glutamate; Gln, glutamine; Cr, creatine; PCr, phosphocreatine; GPC, glycerophosphocholine; Tau, taurine; Gly, glycine; Ans, anserine. Fumarate was also identified on the spectrum and included in all further analyses, but not in the figure because of its large distance to the other peaks. Note significant differences in PGlu and Gln and marked by arrows.

**Fig 3:**
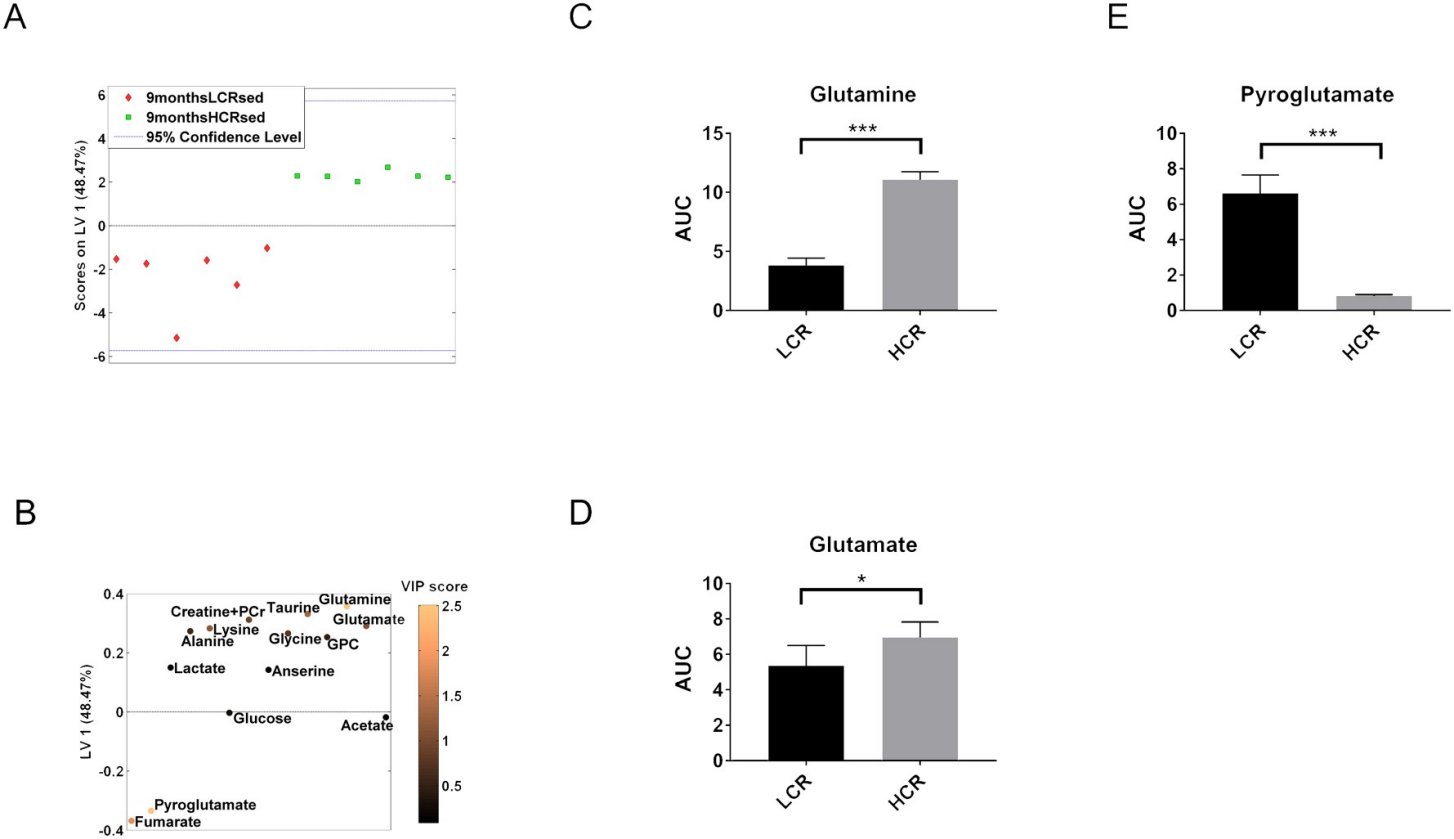
Multivariate and univariate analysis of metabolic profiles from LCR sedentary and HCR sedentary rats at 9 months of age. (**A**) PLS-DA score of sedentary 9 months rats showed a significant difference between LCR sedentary and HCR sedentary rats (permutation test, *P* = 0.04). (**B**) Loading variable 1 (LV1) was used to create the model. Contribution of each metabolite to the model is illustrated by colour, where a lighter shade indicates a greater VIP score and greater contribution to the model. (**C, D, and E**) Pairwise comparison between LCR sedentary and HCR sedentary at 9 months, of glutamine (**C**), glutamate (**D**) and pyroglutamate (**E**). *P* values: *, <0.05; ***, <0.001.

**Fig 4:**
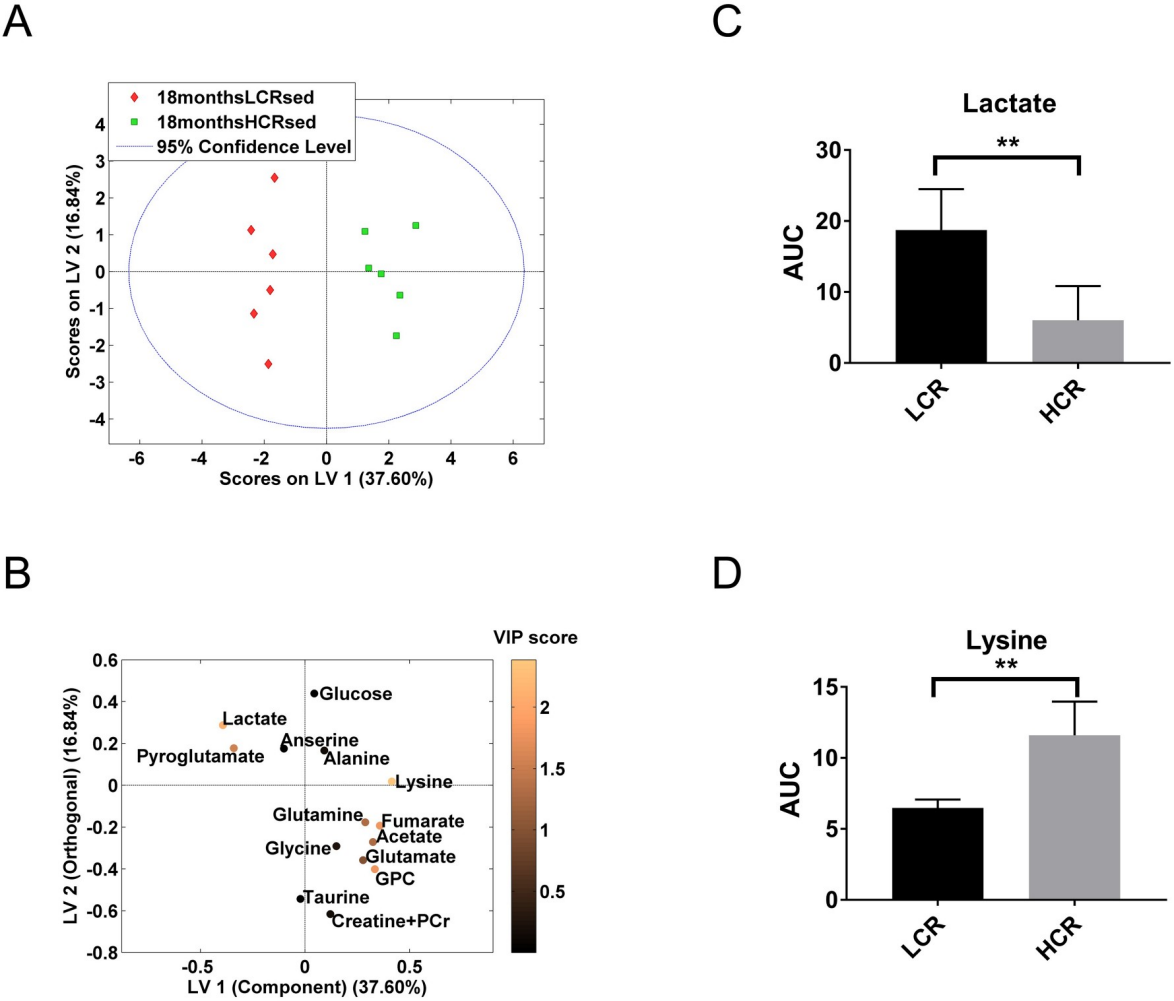
Multivariate and univariate analysis of metabolic profiles from LCR sedentary and HCR sedentary rats at 18 months of age. (**A**) PLS-DA scores acquired from 18 months rats showed a significant difference between LCR sedentary and HCR sedentary (permutation test, *P* = 0.041). (**B**) Loading variables (LV) 1 and 2 were used to create the model. Contribution of each metabolite to the model is illustrated by colour, where a lighter shade indicates a greater VIP score and greater contribution to the model. (**C and D**) Pairwise comparison between LCR sedentary and HCR sedentary at 18 months, of lactate (**C**) and lysine (**D**). *P* value: **, <0.01.

The proton MR spectra from soleus muscle identified 14 metabolites that were used for metabolic profiling (Fig 2, S1 and S2 Figs). First, we compared sedentary LCR rats to their respective HCR counterparts at different ages. At both 9 and 18 months of age, LCR had a significantly different metabolic profile compared to HCR (Table 1, Figs 3 and 4). According to the PLS-DA statistical model, the most important metabolites were glutamine, glutamate and pyroglutamate in the 9 months rats (Fig 3), and lysine and lactate in the 18 months rats (Fig 4). Univariate analysis confirmed that 9 months HCR rats had 2.9 fold, 1.3 fold, and 0.12 fold change in the levels of glutamine, glutamate, and pyroglutamate, respectively (Fig 3, Table 2 and S2 Table). At 18 months of age, HCR rats had 0.32- and 1.8 fold change of lactate and lysine, respectively (Fig 4, Table 3 and S2 Table).

**Table 1:** Summary of PLS-DA model results.

Comparison	Accuracy (%)	Sensitivity (%)	Specificity (%)	Number of LVs	Permutation test *P* value
9 vs. 18-months old rats	93.4	90.9	95.8	5	***P* = 0.001**
LCR sed vs. HCR sed, 9 months	100	100	100	1	***P* = 0.021**
LCR sed vs. HCR sed, 18 months	91.7	100	83.3	2	***P* = 0.041**
LCR sed vs. LCR trained, 9 months	71.7	83.3	60	1	*P* = 0.316
HCR sed vs. HCR trained, 9 months	90	100	80	2	*P* = 0.072
LCR sed vs. LCR trained, 18 months	75	83.3	66.7	3	*P* = 0.269
HCR sed vs. HCR trained, 18 months	83.4	83.3	83.3	1	*P* = 0.267

**Table 2:** Univariate analysis between 9 months LCR sed and 9 months HCR sed.

Metabolites	*P* value	Fold change (HCR_sed/LCR_sed)
Pyroglutamate	< 0.0001	0.12
Fumarate	0.0025	0.4
Acetate	0.53	0.8
Anserine	0.57	1.1
GPC	0.15	1.1
Lactate	0.33	1.2
Alanine	0.14	1.2
Creatine+Phosphocreatine	0.051	1.2
Glycine	0.072	1.2
Glucose	0.32	1.3
Taurine	0.014	1.3
Glutamate	0.022	1.3
lysine	0.02	1.4
Glutamine	< 0.0001	2.9

**Table 3:** Univariate analysis between 18 months LCR sed and 18 months HCR sed.

Metabolites	*P* value	Fold change (HCR_sed/LCR_sed)
Lactate	0.0021	0.32
Pyroglutamate	0.02	0.61
Anserine	0.68	0.93
Glucose	0.91	1
Creatine+Phosphocreatine	0.43	1
Taurine	0.96	1
Alanine	0.46	1.1
Glutamate	0.075	1.1
Glycine	0.37	1.2
GPC	0.0072	1.2
Glutamine	0.026	1.2
Fumarate	0.01	1.3
Acetate	0.032	1.6
lysine	0.0027	1.8

**Fig 5:**
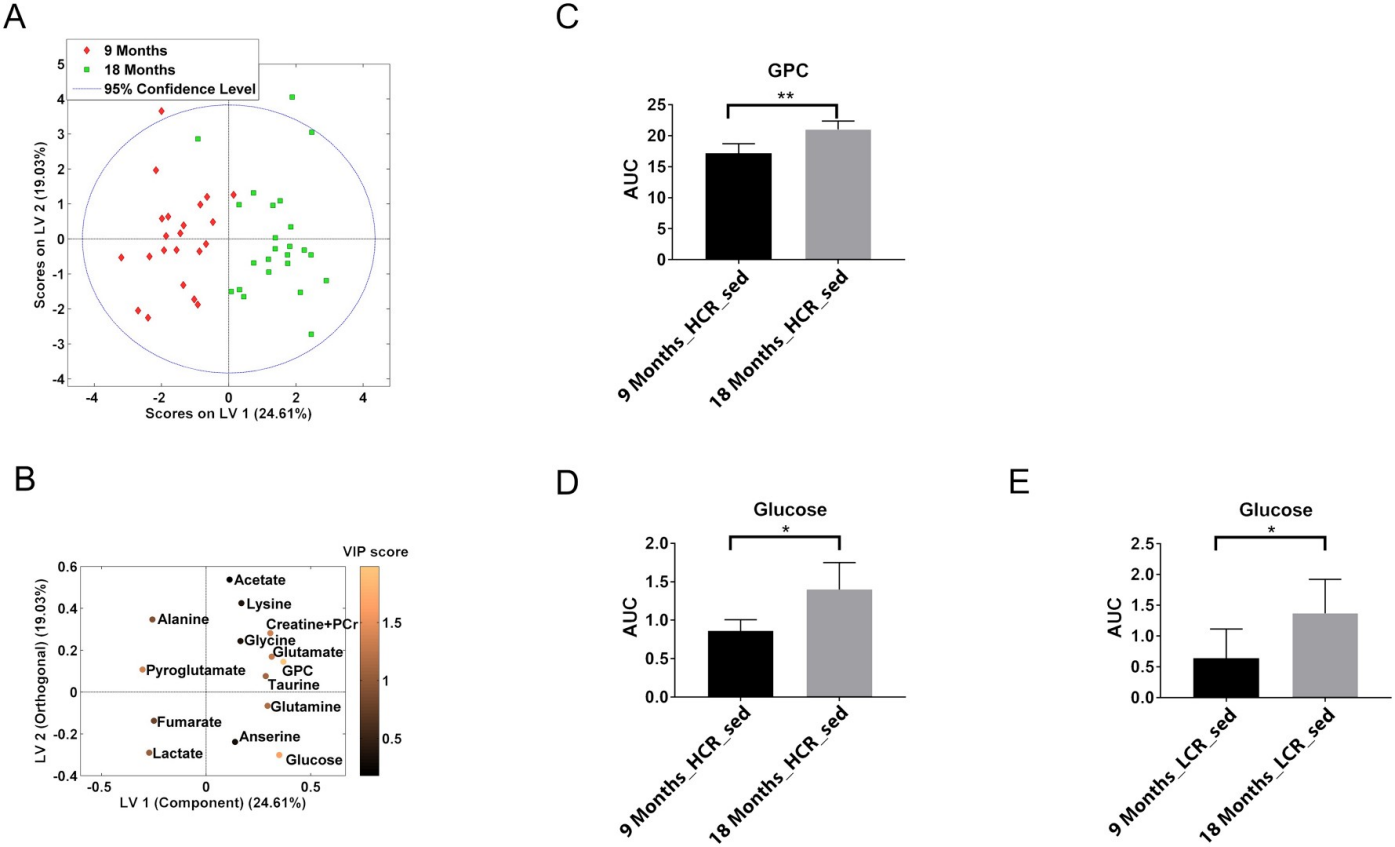
Multivariate and univariate analysis of metabolic profile of 9 months old rats compared to 18 months old rats. (**A**) The score plot of PLS-DA separated 9-month from 18-month (permutation test, *P* = 0.001). (**B**) Loading variables 1 (LV1) and LV2 display the contribution of the individual metabolites to the model. Contribution of each metabolite to the model is illustrated by colour, where a lighter shade indicates a greater VIP score and greater contribution to the model. (**C**) Pairwise comparison between 9 months HCR sed and 18 months HCR sed of glycerophosphocholine (GPC). (**D and E**) Pairwise comparison of glucose between 9 months HCR sed and 18 months HCR sed (**D**) and between 9 months LCR sed and 18 months LCR sed (**E**). *P* values: *, <0.05; **, <0.01.

Next, we determined the general effect of aging on the metabolic profile. Therefore, only aging was fed into the PLS-DA statistical model. The metabolic profile was different between the 9 and 18 months old rats (Table 1, Fig 5A). Glycerophosphocholine and glucose were the most important metabolites according to the VIP scores (Fig 5B). Univariate analysis confirmed that in 18 months old rats, both LCR sed and HCR sed had significantly higher glucose levels than at 9 months of age (2.1 fold in LCR sed and 1.6 fold in HCR sed, respectively). In HCR sed, 18 months old rats had a 1.2 fold higher glycerophosphocholine level compared to 9 months HCR sed. (Fig 5C–5E, Table 4 and S2 Table).

**Table 4:** Univariate analysis between 9 months and 18 months.

	LCR_sed			HCR_sed	
Metabolites	*P* value	fold change (18 months/9 months)	Metabolites	*P* value	fold change (18 months/9 months)
Pyroglutamate	< 0.0001	0.16	Lactate	0.001	0.32
Fumarate	0.008	0.52	Pyroglutamate	0.19	0.77
Acetate	0.58	0.82	Alanine	0.2	0.88
Alanine	0.83	0.97	Anserine	0.89	0.97
Lysine	0.8	1	Taurine	0.93	1
Anserine	0.28	1.1	Glutamine	0.57	1
Glycine	0.25	1.1	Creatine	0.081	1.1
GPC	0.055	1.1	Glycine	0.38	1.2
Lactate	0.38	1.2	GPC	0.001	1.2
Creatine	0.025	1.2	Glutamate	0.038	1.2
Taurine	0.014	1.3	Lysine	0.026	1.3
Glutamate	0.016	1.3	Acetate	0.025	1.6
Glucose	0.035	2.1	Glucose	0.011	1.6
Glutamine	< 0.0001	2.5	Fumarate	0.00074	1.7

The PLS-DA model could not separate the sedentary from the exercise training in any determined groups (S3–S6 Figs), although there was a tendency among the HCRs at 9 months of age (*P* = 0.072, Table 1, and S4 Fig).

## Discussion

The main finding of the present study was that high inborn running capacity was associated with a more favorable metabolic profile in skeletal muscle. HCR rats had higher levels of glutamine and glutamate, and lower levels of lactate compared to LCR, indicating more effective glucose oxidation. Aging was associated with increased glycerophosphocholine and glucose levels in older rats. Exercise training had no effect on metabolic profile. Although running speed was higher in exercising HCR rats, *V*O_2max_ remained unchanged except at 9 months of age. Implications of these observations are discussed below.

The notion that high inborn aerobic capacity is associated with a more favorable metabolic profile is based on the observation that the levels of glutamine and glutamate were higher, and that the level of lactate was lower in soleus muscle from HCR rats than in their LCR counterparts. Even though amino acids contribute moderately to substrate utilization, they are important for the overall energy metabolism [34]. In general, HCR had higher levels of glutamine and glutamate than LCR (Fig 3). Glutamine and glutamate are essential for nitrogen balance and carbohydrate oxidation [35, 36]. Glutamine is derived from glutamate and coupled with pyruvate metabolism and tricarboxylic acid cycle. Thus, higher levels of glutamine and glutamate are consistent with higher levels of energy metabolism.

Glutamine and glutamate are linked to pyroglutamate and pyruvate production. By cyclization, pyroglutamate is formed directly from glutamine and serves as a storage of glutamate. This might explain the decreased level of pyroglutamate in rats with higher glutamate levels (Fig 3). Increased glutamine and glutamate levels may also contribute to pyruvate production. During prolonged submaximal exhaustive exercise, this may prevent fatigue [37].

Lactate played an important role in differentiating the metabolic profile between sedentary HCR and LCR at 18 months of age, with a higher accumulation in the LCR rats even in the aerobic dominant soleus muscle (Fig 4). Lactate production is associated with anaerobic glycolysis from glucose or glycogen [38]. The anaerobic glycolysis maintains energy production when aerobic metabolism is insufficient [39] and is associated with reduced oxidative capacity in LCR [10, 40]. The increased level of lactate in the aged muscles of LCR compared to HCR suggests a decline in mitochondrial function [41], which can result in a more severe metabolic syndrome phenotype with age [1]. Recent studies have demonstrated a positive correlation between lactate levels and prevalence of metabolic syndrome [42].

The significance of increased lysine in 18 months HCR rats (Fig 4) is unclear. Previous studies have demonstrated that oral administration of lysine can suppress myofibrillar protein degradation via the autophagic-lysosomal pathway [43, 44]. However, it is not known whether this effect might be an advantage in aging.

Aging was associated with increased levels of soleus muscle glucose and glycerophosphocholine, independent of inborn aerobic capacity. Glycerophosphocholine is a major component of cell membrane, produced from cell membrane degradation. Similar to our findings, elevated levels of glycerophosphocholine were reported in gastrocnemius muscles from aging rats [45], as well as in overweight and obese rats [46]. Although the underlying mechanism has not been determined, these and several other studies have shown that glycerophosphocholine accumulation is associated with aging, impaired mitochondrial activity, high BMI, and low *V*O_2max_ [45–47].

Aging was also associated with higher skeletal muscle glucose levels (Fig 5). As a major source of energy and carbon, glucose plays an essential role in sustaining the energy metabolism in the muscles. Glucose catabolism is also a supply of metabolic intermediates essential for macromolecular biosynthesis in cell growth and proliferation [48]. Several reports have demonstrated that aging is associated with impaired glucose disposal and insulin sensitivity [49–52]. Insulin response in skeletal muscle can be characterized by 1) oxidative glucose disposal (glucose transportation and oxidation) and 2) non-oxidative glucose disposal (glycogen synthesis) [53] and both are impaired in diabetics, insulin resistant patients and elderly [54, 55]. In a situation where both glycogen synthesis oxidative glucose disposal are impaired, glucose can accumulate in the muscle tissue [56].

## Limitations

A limitation of the present study is that exercise training only provided minor effects on aerobic capacity and running speed, suggesting that the exercise volume was too low to induce robust changes in metabolic profile. The rationale behind the low training volume was that clinical and epidemiological studies have shown significantly improved outcomes with only minor changes in *V*O_2max_ or going from sedentary to low levels of physical activity [3, 57–59]. Hence, the lack of robust effects on the skeletal muscle metabolic profile does not preclude beneficial health effects.

Only soleus muscle was used in our study, which might not fully represent all skeletal muscle metabolic profile. In rats, the soleus is an aerobic skeletal muscle with mainly type 1 muscle fibers and high oxidative capacity, and one might indicate that the high aerobic capacity in the soleus could mask adaptation to environmental changes. However, muscle fiber type has been reported to be different in mixed and glycolytic muscles between HCR and LCR but not in soleus. This means that muscle fiber type distribution between HCR and LCR would probably not bias the responses observed in the study.

## Conclusion

Soleus muscle from rats with high intrinsic running capacity showed higher levels of glutamine and glutamate and lower levels of lactate, indicating more efficient glucose oxidation. During aging, the levels of glycerophosphocholine and glucose were upregulated. Differences in metabolic profile were associated with the differing intrinsic exercise capacity as well as aging, and correlated to *V*O_2max_.

## Supporting information

S1 Fig Proton MR spectra in soleus muscle from sedentary rats at 18 months of age. Spectra in figure show mean values of all measurements in either group. Upper panel: HCR; Lower panel LCR. Metabolite labeling: Lac, lactate; Ala, alanine; Lys, lysine; Ace, acetate; Glu, glutamate; Gln, glutamine; Cr, creatine; PCr, phosphocreatine; GPC, glycerophosphocholine; Tau, taurine; Gly, glycine; Ans, anserine. Fumarate was also identified on the spectrum and included in all further analyses, but not in the figure because of its large distance to the other peaks. Note significant differences in Lac and Lys.
(TIF)

S2 Fig Proton MR spectra in soleus muscle from all samples. Spectra in figure show mean values of all measurements in either group. Upper panel: 9 months; Lower panel: 18 months. Metabolite labeling: Lac, lactate; Ala, alanine; Lys, lysine; Ace, acetate; Glu, glutamate; Gln, glutamine; Cr, creatine; PCr, phosphocreatine; GPC, glycerophosphocholine; Tau, taurine; Gly, glycine; Ans, anserine. Fumarate was also identified on the spectrum and included in all further analyses, but not in the figure because of its large distance to the other peaks. Note significant differences in GPC and Glu.
(TIF)

S3 Fig Multivariate analysis of metabolic profiles from 9 months LCR sedentary group compared to training groups. **Panel A:** PLS-DA score plot. **Panel B:** Loading plot for all metabolites.
(TIF)

S4 Fig Multivariate analysis of metabolic profiles from 9 months HCR sedentary group compared to training groups. **Panel A:** PLS-DA score plot. **Panel B:** Loading plot for all metabolites.
(TIF)

S5 Fig Multivariate analysis of metabolic profiles from 18 months LCR sedentary group compared to training groups. **Panel A:** PLS-DA score plot. **Panel B:** Loading plot for all metabolites.
(TIF)

S6 Fig Multivariate analysis of metabolic profiles from 18 months HCR sedentary group compared to training groups. **Panel A:** PLS-DA score plot. **Panel B:** Loading plot for all metabolites.
(TIF)

S1 Table Original data of *V*O_2max_ and running speed. (XLSX)

S2 Table Original data of integration of all metabolites. (XLSX)
