# Supplementary figures and images for: Skeletal muscle metabolism in rats with low and high intrinsic aerobic capacity: Effect of aging and exercise training
Source: PLoS One. 2018 Dec 11;13(12):e0208703. doi: 10.1371/journal.pone.0208703 (PMC6289443; doi:10.1371/journal.pone.0208703)

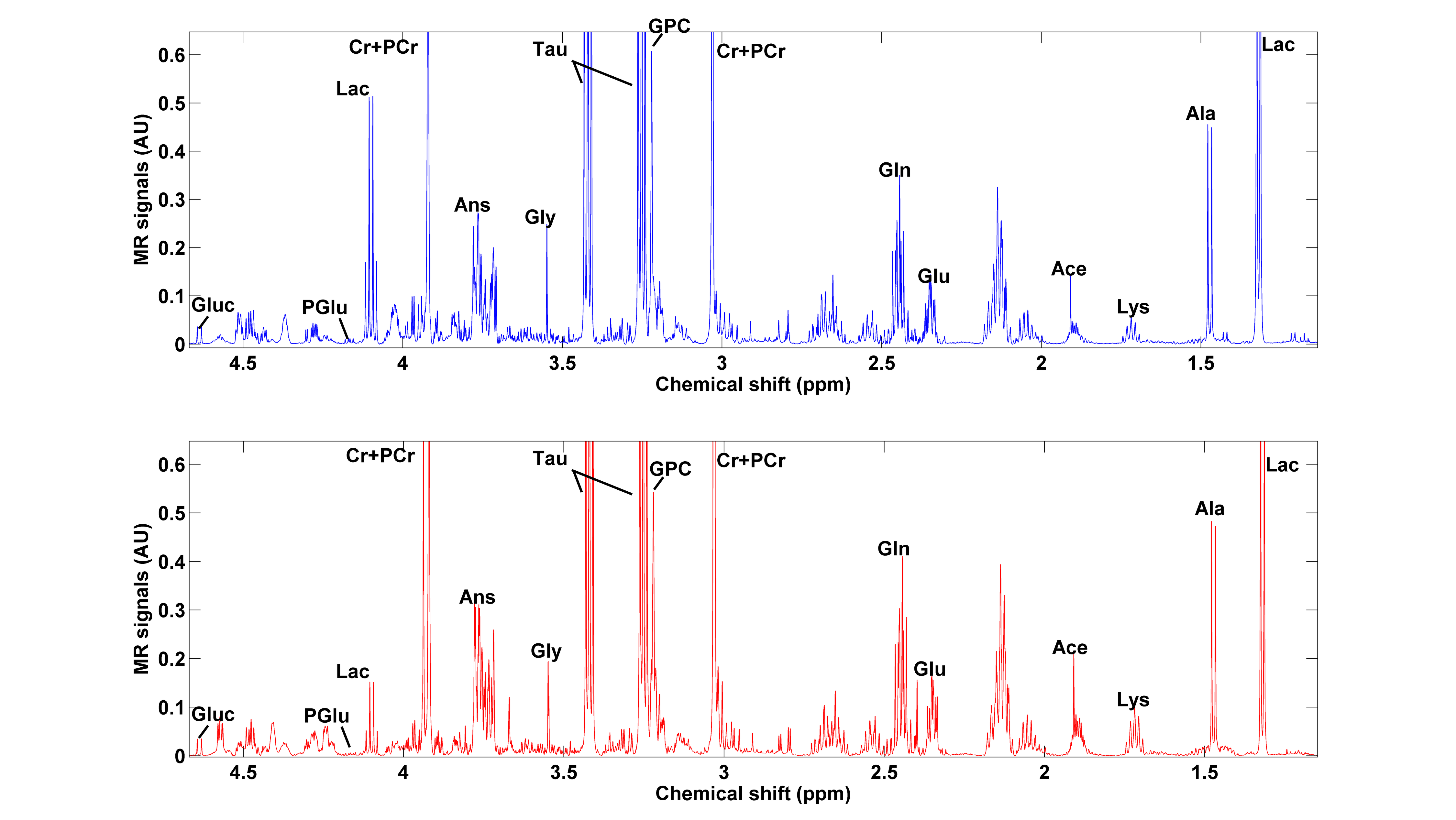

Supplement: S1 Fig — Spectra in figure show mean values of all measurements in either group. Upper panel: HCR; Lower panel LCR. Metabolite labeling: Lac, lactate; Ala, alanine; Lys, lysine; Ace, acetate; Glu, glutamate; Gln, glutamine; Cr, creatine; PCr, phosphocreatine; GPC, glycerophosphocholine; Tau, taurine; Gly, glycine; Ans, anserine. Fumarate was also identified on the spectrum and included in all further analyses, but not in the figure because of its large distance to the other peaks. Note significant differences in Lac and Lys. (TIF) [file pone.0208703.s001.tif]

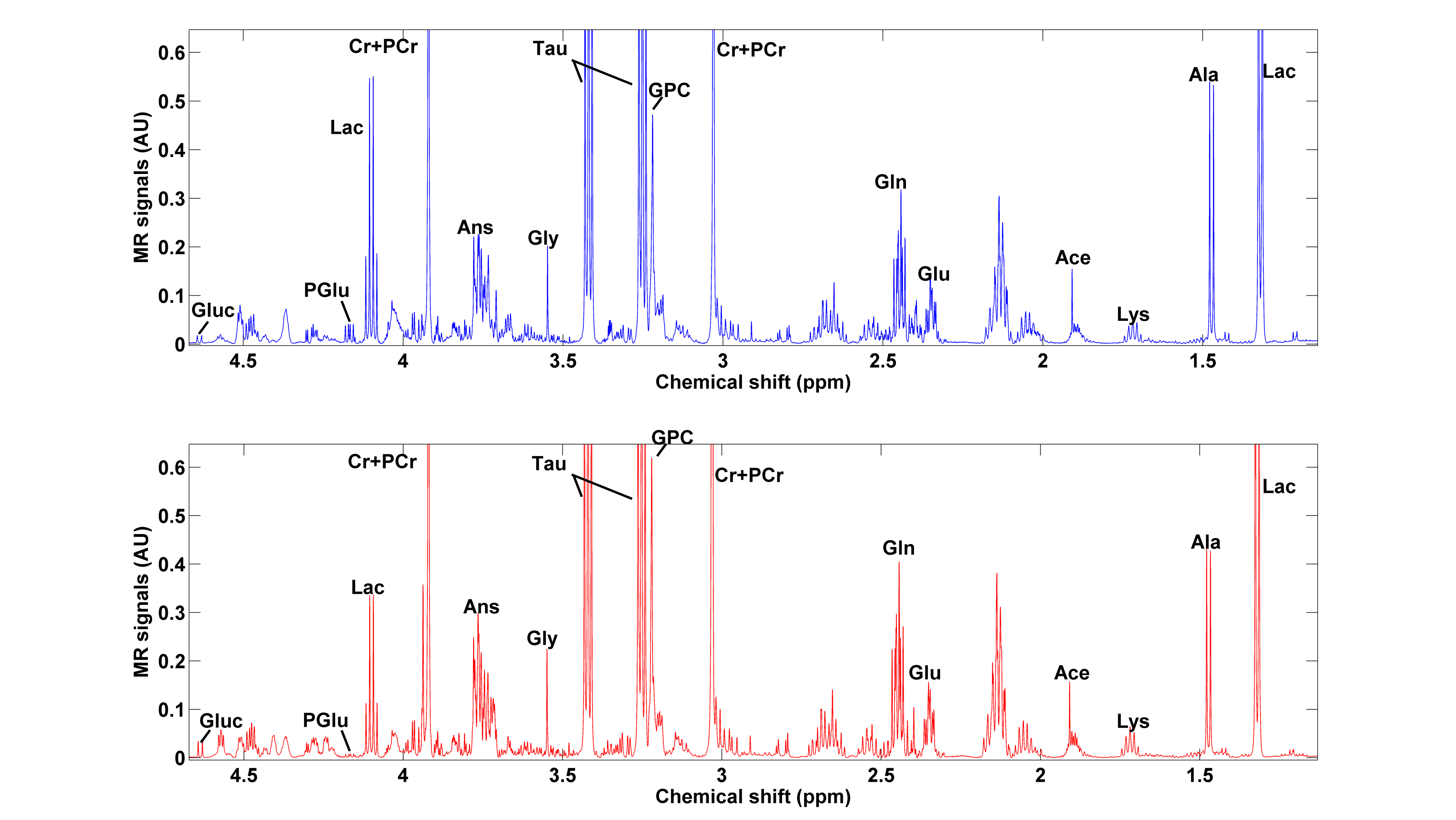

Supplement: S2 Fig — Spectra in figure show mean values of all measurements in either group. Upper panel: 9 months; Lower panel: 18 months. Metabolite labeling: Lac, lactate; Ala, alanine; Lys, lysine; Ace, acetate; Glu, glutamate; Gln, glutamine; Cr, creatine; PCr, phosphocreatine; GPC, glycerophosphocholine; Tau, taurine; Gly, glycine; Ans, anserine. Fumarate was also identified on the spectrum and included in all further analyses, but not in the figure because of its large distance to the other peaks. Note significant differences in GPC and Glu. (TIF) [file pone.0208703.s002.tif]

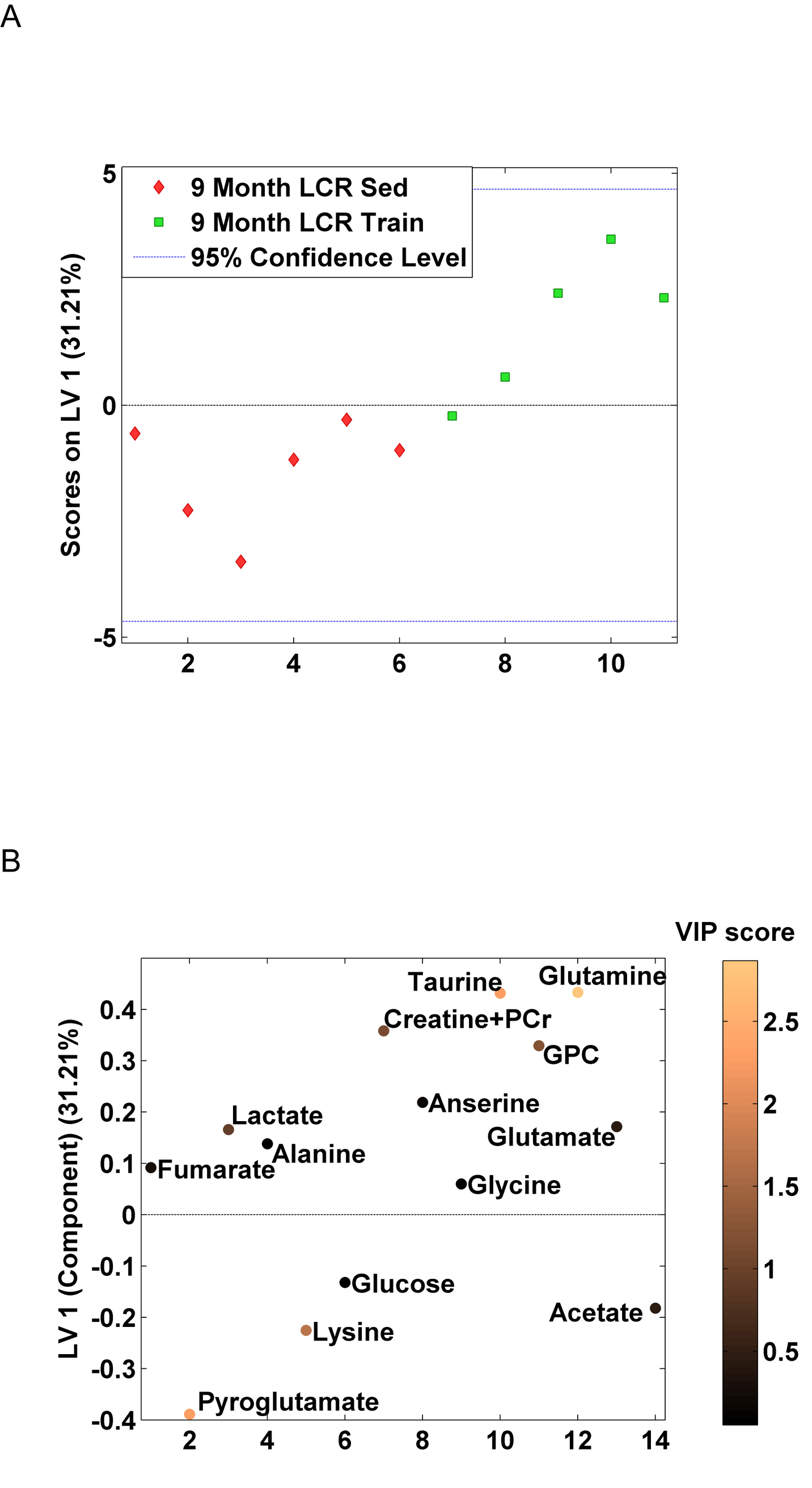

Supplement: S3 Fig — Panel A: PLS-DA score plot. Panel B: Loading plot for all metabolites. (TIF) [file pone.0208703.s003.tif]

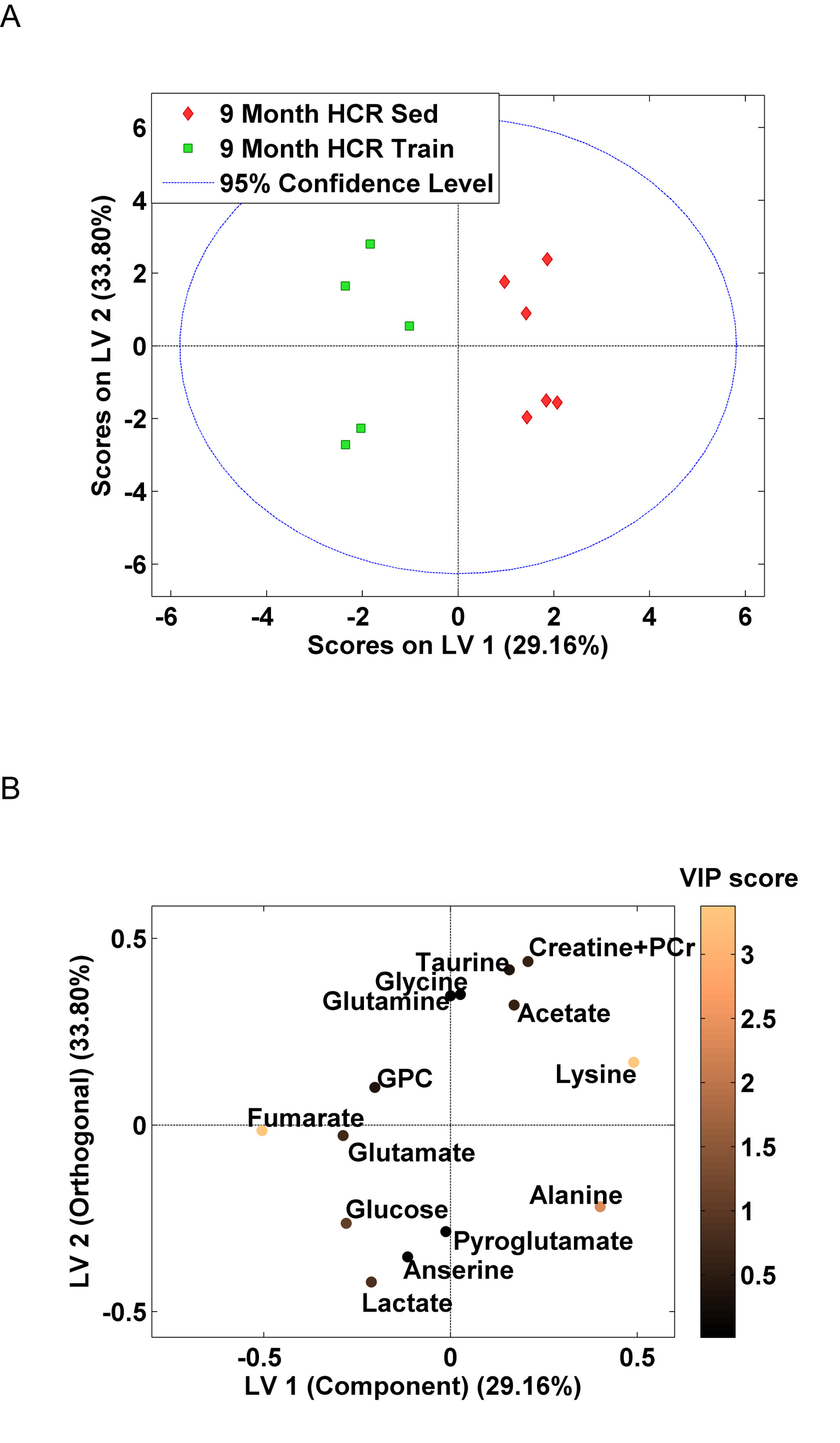

Supplement: S4 Fig — Panel A: PLS-DA score plot. Panel B: Loading plot for all metabolites. (TIF) [file pone.0208703.s004.tif]

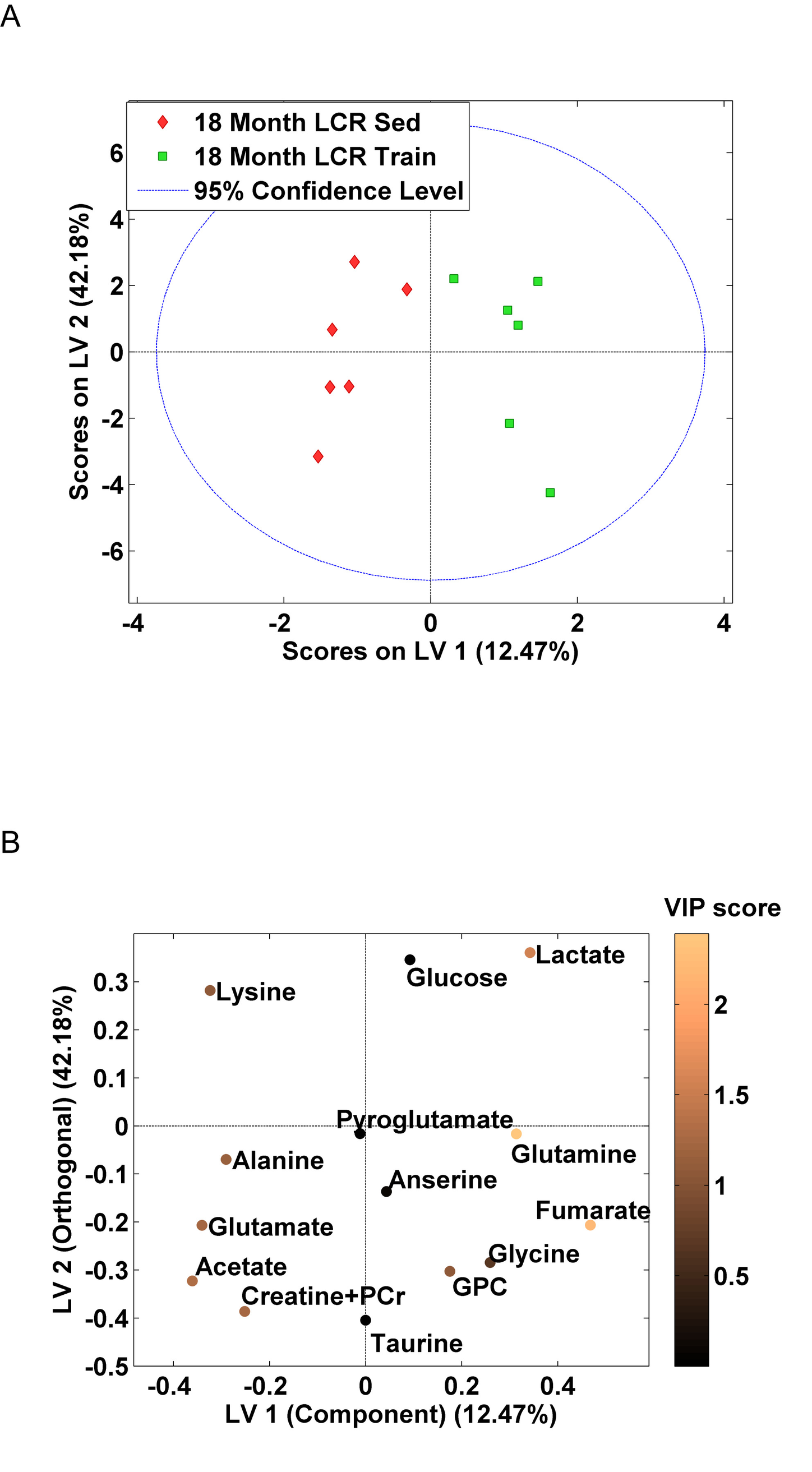

Supplement: S5 Fig — Panel A: PLS-DA score plot. Panel B: Loading plot for all metabolites. (TIF) [file pone.0208703.s005.tif]

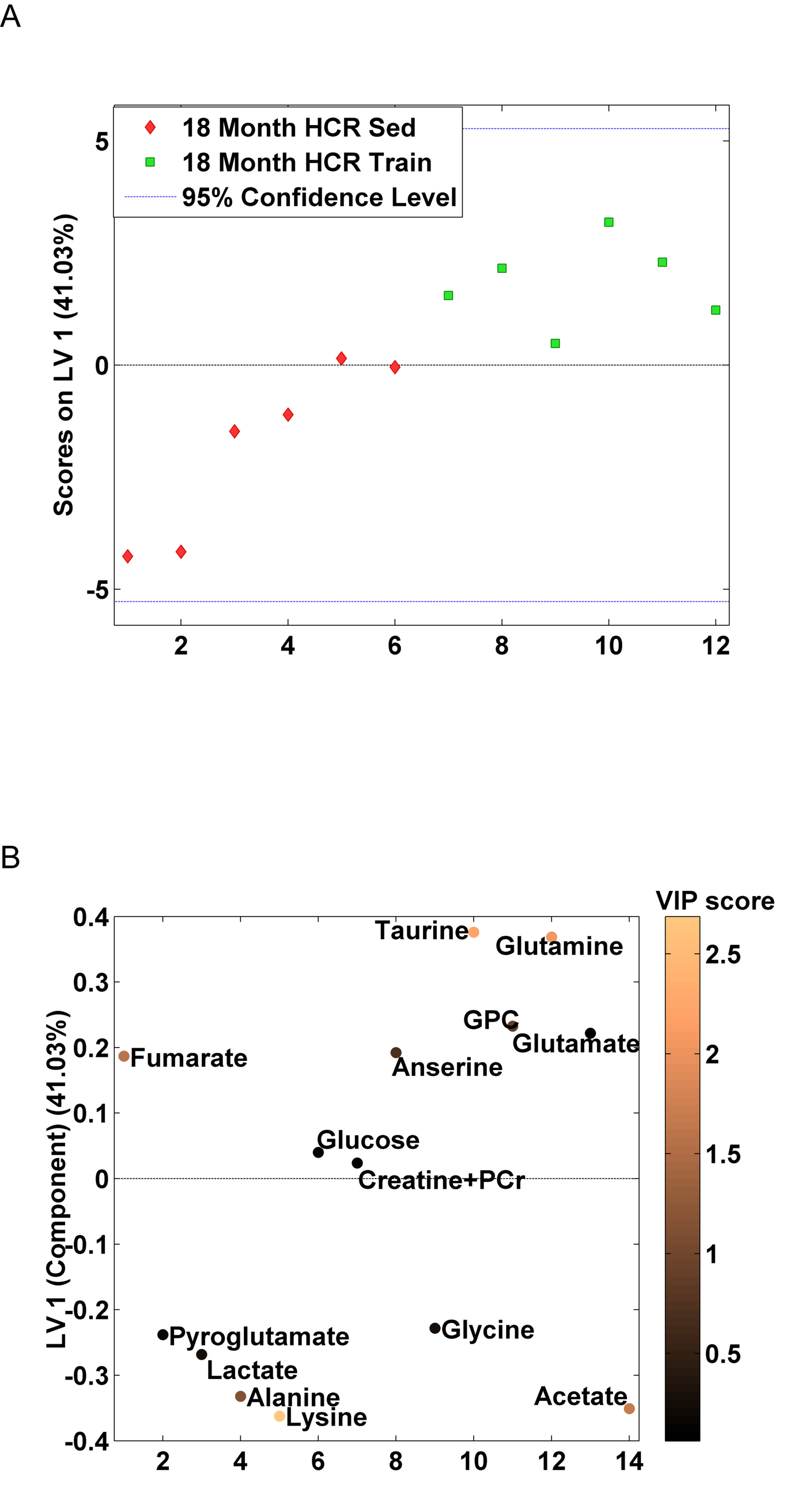

Supplement: S6 Fig — Panel A: PLS-DA score plot. Panel B: Loading plot for all metabolites. (TIF) [file pone.0208703.s006.tif]
